# Dental Formulary

**Published:** 1911-11-15

**Authors:** 


### BIBLIOGRAPHY.

DENTAL FORMULARY, by Herman Prinz, M.D., D.D.S., and published by Lee Smith & Son Co., of Pittsburgh. This, the second edition of Dr. Prinz's manual of Dental Formulae is much improved, as it has been thoroughly revised or practically rewritten. This work should be in the hands of every practitioner as it contains practically every useful formula known at the present time which has any merit. The author has taken great pains to select only reliable formula, and because of his familiarity with dental drugs, &c. he is wonderfully capable of selecting only such as would merit his expert approval. Few men of Dr. Prinz's capacity would take the time or have the patience to look up the literature for so varied an exposition as he has compressed into this small volume of 350 pages. The book is well printed and made up and can be had of all Dental Depots.

